# Convolutional neural networks for prostate cancer detection, classification, and segmentation: A systematic review and bibliometric analysis

**DOI:** 10.1016/j.ejro.2026.100741

**Published:** 2026-03-03

**Authors:** Burak Gülmez

**Affiliations:** a College of Engineering and Architecture, University College Dublin, Dublin, Ireland; b Veta Apt, Dumlupinar Mah. Anafartalar Cad. Cil Sok. No: 3, 16285, Ni̇lufer, Bursa, Türkiye

**Keywords:** Convolutional neural networks, Prostate cancer, Deep learning, Medical imaging, Computer-aided diagnosis, Systematic review

## Abstract

**Background:**

Prostate cancer represents the second most common malignancy among men globally, necessitating accurate diagnostic methodologies for optimal patient outcomes. Convolutional neural networks (CNNs), a core deep learning methodology, have emerged as transformative technologies for automated prostate cancer detection, classification, and segmentation across multiple imaging modalities.

**Materials and methods:**

A systematic review following PRISMA guidelines was conducted across Web of Science, Scopus, and PubMed databases (January 2020–December 2025). CNN-based classification architectures were analyzed across ResNet, Vision Transformer, DenseNet, Xception, ConvNeXT, and Swin Transformer implementations, with comparative evaluation of accuracy and transfer learning performance. Object detection and segmentation approaches were examined across U-Net variants, R-CNN family algorithms, and YOLO-based implementations. Hyperparameter optimization strategies were assessed. Explainable AI methodologies including SHAP, Grad-CAM, DiCE, and LIME were evaluated for clinical interpretability and spatial localization accuracy.

**Results:**

Analysis of 320 publications revealed peak research activity in 2024 (63 publications, 19.7%). The United States led with 58 publications (18.1%), followed by China with 55 (17.2%). Multiparametric MRI constituted the primary imaging modality (42.5%), followed by histopathology (28.1%), ultrasound (14.1%), and PET imaging (9.4%). Vision Transformer models demonstrated the highest classification accuracy among evaluated architectures, while U-Net variants dominated segmentation applications with consistently high Dice coefficients. SHAP emerged as the most frequently adopted explainability method across the reviewed studies.

**Conclusions:**

CNN-based prostate cancer detection, classification, and segmentation demonstrate promise for improving diagnostic accuracy and clinical workflow efficiency, though challenges in dataset standardization, regulatory compliance, and clinical integration remain to be addressed.

## Highlights

• Comprehensive analysis of publications reveals CNN advancement in prostate cancer detection.
• Vision Transformers achieve high accuracy while U-Net dominates segmentation applications.
• SHAP explainability methods appear in huge ratio of studies addressing clinical interpretability.
• Dataset heterogeneity and computational constraints limit widespread clinical translation.
• Multi-modal integration and federated learning present promising future research directions.

## 1 Introduction

Prostate cancer represents the second most frequently diagnosed malignancy among men worldwide, with approximately 1.4 million new cases reported annually [1], [2]. The disease exhibits complex heterogeneity in its clinical presentation, ranging from indolent lesions requiring active surveillance to aggressive forms demanding immediate intervention.

The diagnostic workflow for prostate cancer relies on a multimodal approach. Digital rectal examination serves as the initial screening tool, though its sensitivity remains limited, detecting only palpable lesions that have progressed beyond early stages. Prostate-specific antigen (PSA) testing provides a biochemical marker, yet high false-positive rates (approximately 70% for PSA values between 4 and 10 ng/mL) lead to unnecessary biopsies and patient anxiety [3], [4], [5]. Transrectal ultrasound-guided biopsy, the standard confirmatory procedure, suffers from sampling errors, with systematic biopsies missing clinically significant cancers in approximately 20–30% of cases [6], [7].

Multiparametric magnetic resonance imaging (mpMRI), now established as a standard diagnostic methodology, integrates T2-weighted, diffusion-weighted, and dynamic contrast-enhanced sequences to improve lesion detection and characterization. The PI-RADS scoring system has standardized mpMRI reporting, yet reader variability persists, with inter-reader agreement for PI-RADS scores reported as moderate (kappa 0.46–0.78) [8], [9]. Additionally, mpMRI demonstrates reduced sensitivity for lesions smaller than 10 mm and those located in the transition zone. Histopathological assessment through Gleason grading remains subject to inter-observer variability ranging from 10% to 25% across pathologists [10], [11]. Positron emission tomography (PET) imaging, particularly with PSMA tracers, provides molecular-level information but involves radiation exposure and limited spatial resolution for small lesions.

The emergence of artificial intelligence and deep learning methodologies has introduced automated approaches to address these technique-specific limitations. CNNs have shown capability in medical image analysis tasks, achieving diagnostic accuracy comparable to expert clinicians in certain applications [12].

CNN architectures have evolved through distinct generations, each addressing specific computational challenges. Early implementations relied on standard convolutional layers for feature extraction, exemplified by VGG and ResNet architectures that established baseline performance benchmarks. Recent studies have applied various CNN architectures for prostate cancer analysis, including U-Net variants for segmentation tasks [13], [14], and Vision Transformers for classification applications [15], [16]. Object detection algorithms such as YOLO-based implementations [17] and R-CNN variants [18] have shown capability in identifying suspicious regions.

The transition from pure convolutional approaches to attention-based architectures represents a natural progression: while CNNs capture local spatial patterns effectively, transformer models address the need for global context understanding in medical images. Hybrid architectures combining CNN spatial processing with transformer attention mechanisms have emerged as a compromise, retaining local feature extraction while incorporating long-range dependencies [19], [20].

Building effective deep learning models requires systematic hyperparameter optimization, which has progressed from manual tuning toward automated approaches. Genetic algorithms [21] and Bayesian optimization strategies [22] have shown improved model performance. As these models gain complexity, the need for interpretability in clinical settings has driven the adoption of explainable AI (XAI) methodologies. SHAP [19], [20] and Grad-CAM implementations [14], [23] provide interpretability insights for clinical decision-making.

Despite these advances, persistent challenges limit clinical translation of CNN-based prostate cancer systems. Dataset heterogeneity, imaging protocol variations, and limited generalizability across institutions remain obstacles, as documented in recent systematic evaluations of radiomics studies in prostate cancer [24]. Additionally, methodological quality concerns have been raised regarding the standardization of AI-based medical imaging studies [25].

This systematic review aims to analyze CNN applications for prostate cancer detection, classification, and segmentation, examining architectural approaches, dataset characteristics, performance metrics, and clinical translation potential across publications from 2020 to 2025.

## 2 Materials and methods

This systematic review employed a search strategy across multiple academic databases to identify relevant publications addressing CNN applications in prostate cancer detection, classification, and segmentation. The methodology followed PRISMA guidelines to ensure systematic identification, screening, and analysis of the literature.

### 2.1 Search strategy

The literature search encompassed three primary databases: Web of Science (WoS), Scopus, and PubMed, covering the period from January 2020 to December 2025. The last search was conducted on December 15, 2025. The search strategy utilized Boolean operators and controlled vocabulary terms to maximize retrieval of relevant publications while maintaining precision.

Search terms included combinations of the following keywords: “prostate cancer,” “convolutional neural network,” “CNN,” “deep learning,” “artificial intelligence,” “machine learning,” “medical imaging,” “MRI,” “ultrasound,” “histopathology,” “classification,” “segmentation,” “detection,” and “diagnosis.” Database-specific syntax adaptations ensured equivalent search coverage across platforms.

### 2.2 Inclusion and exclusion criteria

Inclusion criteria:

• Peer-reviewed research articles and conference proceedings published between 2020 and 2025
• Studies focusing on CNN applications for prostate cancer detection, classification, or segmentation
• Publications reporting quantitative performance metrics
• Articles published in English language
• Studies utilizing medical imaging data (MRI, ultrasound, CT, histopathology, PET)

Conference proceedings were included alongside peer-reviewed journal articles to capture emerging methodologies and recent architectural innovations that may not yet appear in journal publications. Conference venues such as SPIE Medical Imaging and IEEE conferences represent primary dissemination channels for novel deep learning approaches in medical imaging, and their exclusion would risk overlooking timely contributions to the field.

Exclusion criteria:

• Review articles, editorials, and opinion pieces
• Studies not specifically addressing prostate cancer applications
• Publications lacking quantitative evaluation metrics
• Non-English publications
• Duplicate publications across databases

### 2.3 Study selection process

Initial database searches across Scopus, Web of Science, and PubMed yielded 1,247 records, with 15 additional records identified through manual reference searching. Title and abstract screening excluded 643 records based on irrelevance to CNN-based prostate cancer applications.

Full-text review of 555 articles resulted in final inclusion of 320 publications meeting all selection criteria. Reasons for exclusion during full-text review included studies not employing CNN-based methodologies (n = 98), scope outside prostate cancer applications (n = 62), review articles and meta-analyses (n = 45), and unavailability of full text (n = 30).

**Fig. 1:**
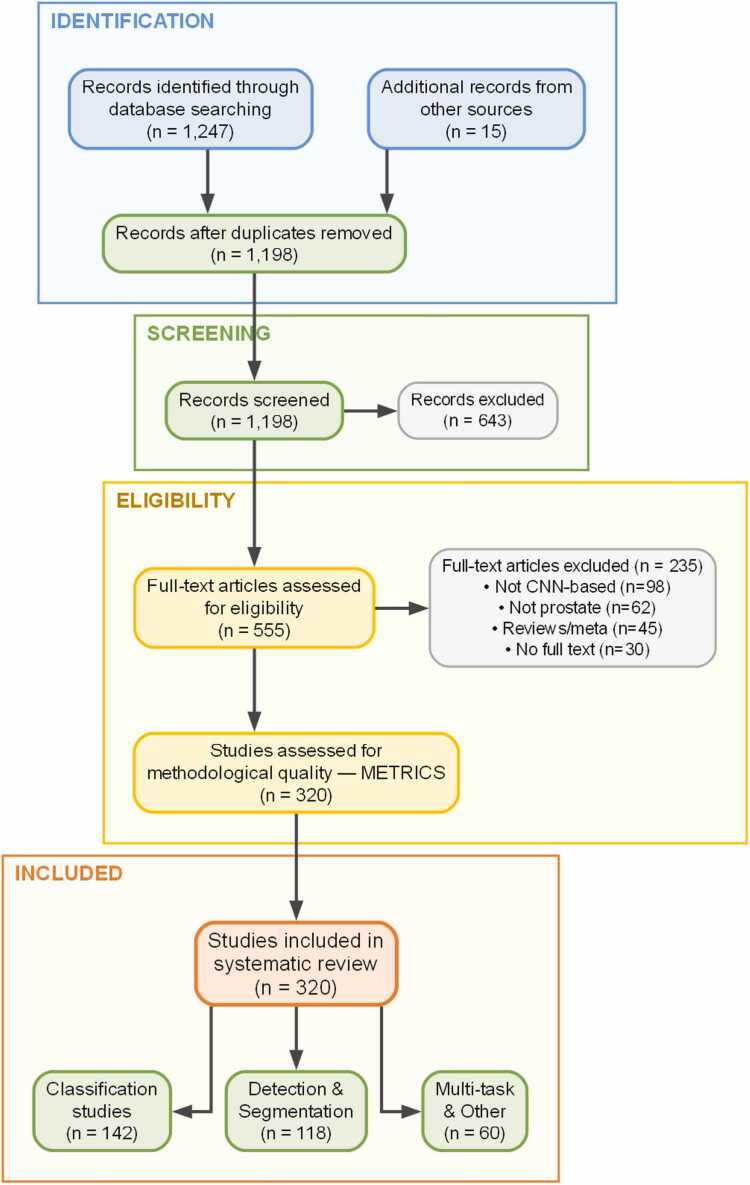
PRISMA flowchart of the study selection process. PRISMA flowchart of the study selection process.

The PRISMA flowchart (Fig. 1) illustrates the progressive screening process from initial identification through final inclusion.

### 2.4 Data extraction

Structured data extraction captured the following information from each included study:

• Bibliographic details (authors, title, journal, year, DOI)
• Study characteristics (dataset size, imaging modality, patient population)
• Algorithmic approaches (architecture type, optimization methods, evaluation metrics)
• Technical specifications (hardware requirements, processing time, validation approach)
• Performance outcomes (accuracy, sensitivity, specificity, Dice coefficient)
• Clinical applications and validation approaches

### 2.5 Quality assessment

Study quality assessment was performed using adapted criteria from the METRICS (Minimum Essential Transparent Reporting Items for Clinical Studies) framework proposed by the European Society of Imaging Informatics [25]. The METRICS tool provides standardized quality evaluation criteria specifically designed for AI-based medical imaging studies, addressing study design, data handling, model development, and reporting completeness.

Assessment criteria included:

• Study design and data source transparency
• Dataset characteristics and size adequacy
• Data preprocessing and augmentation reporting
• Validation methodology rigor
• Performance metric appropriateness
• Reference standard definition
• Clinical relevance and applicability
• Methodological reproducibility

Each study was evaluated against the METRICS criteria, and quality scores informed the synthesis of findings, with particular attention to studies meeting higher quality thresholds.

### 2.6 Analysis approach

The analysis employed both quantitative and qualitative synthesis approaches. Bibliometric analysis characterized publication trends, geographical distribution, and research evolution patterns. Algorithmic analysis categorized approaches by architecture type, application domain, and performance characteristics.

Statistical synthesis proved challenging due to heterogeneity in datasets, evaluation protocols, and performance metrics across studies. Narrative synthesis approaches summarized findings within categorical frameworks while identifying patterns and trends across the literature.

## 3 Results

### 3.1 Study selection

The systematic search identified 1,247 records across Scopus, Web of Science, and PubMed, supplemented by 15 additional records from manual sources. After removing 64 duplicates, 1,198 unique citations underwent title and abstract screening, resulting in exclusion of 643 records. Full-text assessment of 555 articles led to final inclusion of 320 publications (Fig. 1). The PRISMA flowchart details the progressive screening at each stage.

### 3.2 Bibliometric analysis

#### 3.2.1 Temporal publication trends

**Fig. 2:**
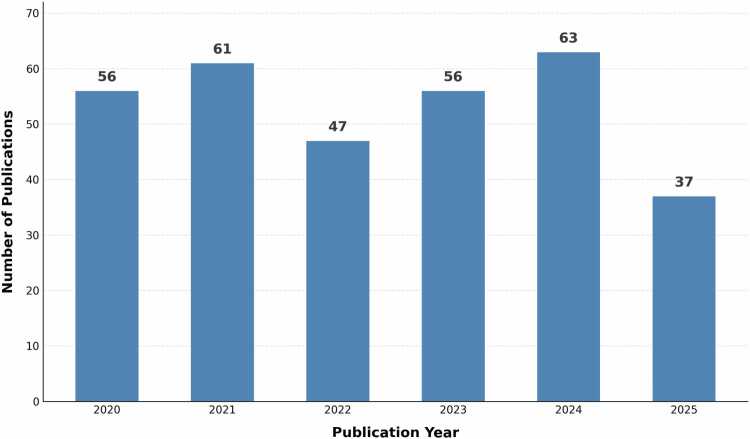
Annual publication trends, 2020–2025. Annual publication trends, 2020–2025.

Fig. 2 illustrates the annual publication distribution across the study period. The analysis reveals fluctuating research activity, with 2024 representing the peak publication year (63 publications, 19.7%), followed by 2021 (61 publications, 19.1%). Publication volumes in 2020 and 2023 remained comparable (56 publications, 17.5% each), while 2022 (47 publications, 14.7%) and 2025 (37 publications, 11.6%) represent moderate activity levels.

#### 3.2.2 Geographical distribution

**Fig. 3:**
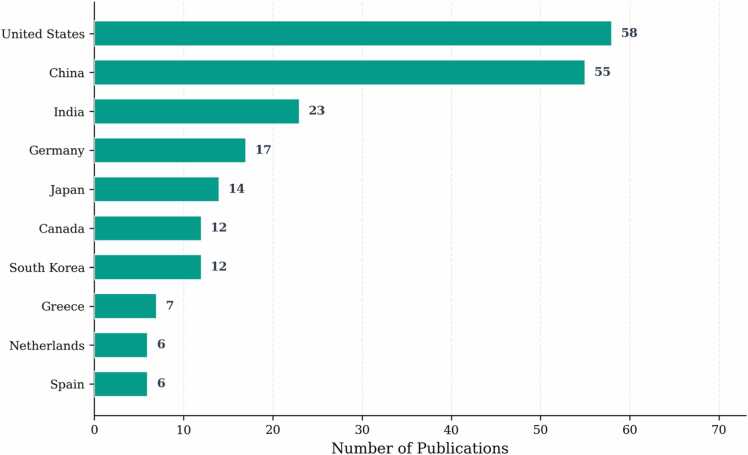
Top 10 countries by publication count. Top 10 countries by publication count.

The geographical analysis reveals concentrated research activity across specific regions. Fig. 3 presents the top 10 contributing countries by publication volume. The United States leads with 58 publications (18.1%), followed by China with 55 publications (17.2%). India contributes 23 publications (7.2%), with the remaining top contributors including Germany (17, 5.3%), Japan (14, 4.4%), Canada (12, 3.8%), South Korea (12, 3.8%), Greece (7, 2.2%), Netherlands (6, 1.9%), and Spain (6, 1.9%). Geographic concentration in North America, Asia, and Europe reflects established medical imaging research infrastructure and healthcare technology investment patterns.

#### 3.2.3 Publication venue and type analysis

**Fig. 4:**
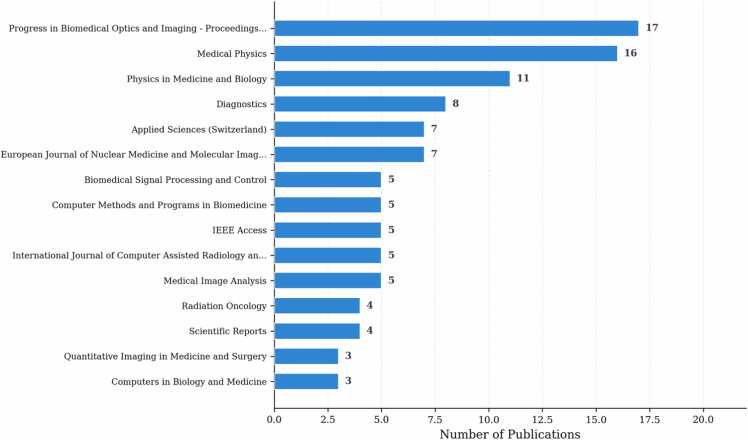
Distribution across top 15 academic venues. Distribution across top 15 academic venues.

Fig. 4 presents the distribution across academic journals and conference proceedings. Progress in Biomedical Optics and Imaging - Proceedings of SPIE emerges as the leading publication venue with 17 publications (5.3%), followed by Medical Physics with 16 publications (5.0%). Physics in Medicine and Biology contributes 11 publications (3.4%), while Diagnostics, Applied Sciences, and European Journal of Nuclear Medicine and Molecular Imaging each contribute 7–8 publications.

The analyzed corpus comprises 285 journal articles (89.1%) and 35 conference proceedings (10.9%). Medical imaging and physics journals represent the primary publication channels, with the venue diversity reflecting the interdisciplinary nature of the field spanning computer science, medical physics, and clinical radiology.

#### 3.2.4 Keyword analysis

**Fig. 5:**
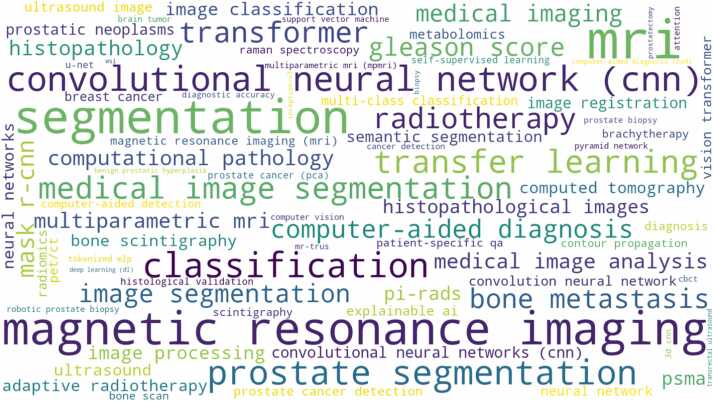
Word cloud of dominant keywords.Word cloud of dominant keywords.

The word cloud analysis (Fig. 5) reveals dominant research themes within the examined literature. “Deep learning,” “CNN,” and “neural network” appear as primary methodological keywords, while “prostate cancer,” “segmentation,” and “classification” represent core application areas. Medical imaging modality terms including “MRI,” “histopathology,” and “ultrasound” indicate multi-modal research activity.

Technical approaches are evidenced by “U-Net,” “transfer learning,” “machine learning,” and “artificial intelligence” terms. Clinical applications emerge through “diagnosis,” “detection,” “radiotherapy,” and “brachytherapy” keywords. The prominence of “PSMA,” “histopathology,” and “Gleason” terms indicates disease-specific analytical focus within the CNN research community.

The keyword analysis reveals evolution from traditional machine learning approaches toward deep learning methodologies, with increasing emphasis on explainable AI and clinical translation. The prominence of specific architectural terms (U-Net, ResNet, Vision Transformer) indicates architectural diversification across applications.

#### 3.2.5 Imaging modality distribution

The distribution of publications by imaging modality reveals that multiparametric MRI constitutes the most frequently investigated data type (136 publications, 42.5%), followed by histopathological imaging (90 publications, 28.1%), ultrasound (45 publications, 14.1%), PET imaging (30 publications, 9.4%), and other modalities including CT and cone-beam CT (19 publications, 5.9%). Several studies employed multiple imaging modalities simultaneously. Public benchmark datasets referenced across the literature include PROMISE12 [26], [27] for prostate segmentation in MRI and the PROSTATEx Challenge [26], [27] for clinically significant prostate cancer detection, both serving as standardized evaluation benchmarks enabling cross-study comparison.

## 4 Literature review

### 4.1 Dataset analysis

The examination of datasets employed across CNN-based prostate cancer detection studies reveals patterns in data acquisition, imaging modalities, and dataset characteristics. Analysis of 320 publications indicates predominant reliance on medical imaging datasets spanning multiple modalities and institutional sources.

Magnetic resonance imaging constitutes the primary data source across reviewed studies, with multiparametric MRI (mpMRI) sequences including T2-weighted, diffusion-weighted, and dynamic contrast-enhanced acquisitions [19], [28], [29]. Histopathological whole-slide images represent the second most common data type across classification studies [30], [31], [32].

Ultrasound imaging datasets appear across multiple studies, particularly for real-time guidance applications and transrectal ultrasound-guided procedures [15], [33]. Positron emission tomography datasets utilizing PSMA tracers show increasing utilization for molecular-level prostate cancer characterization [34], [35].

Dataset sizes exhibit considerable variation, ranging from small institutional cohorts to large multi-center collections. Public dataset availability remains limited, with most studies relying on proprietary institutional data. The PROMISE12 challenge dataset [26], [27] appears in multiple publications, serving as standardized benchmarks for segmentation performance comparison, while the PROSTATEx Challenge dataset [26], [27] provides standardized evaluation for detection tasks.

Image preprocessing approaches show standardization across studies, including intensity normalization, spatial resampling, and augmentation techniques. Data augmentation strategies encompass geometric transformations, intensity variations, and synthetic image generation to address dataset size limitations.

Table 1 presents a systematic analysis of dataset characteristics extracted from the reviewed literature, categorizing imaging modalities, sample sizes, availability patterns, main findings, and study strengths and limitations.

**Table 1:** Dataset analysis across reviewed studies.

Dataset Type	Representative Studies	Image Count Range	Modality	Public Availability	Main Findings	Strengths	Limitations
Multiparametric MRI	Li et al. [19], Rippa et al. [28]	500–2000	T2W, DWI, DCE	Limited	Most widely used modality (42.5%); multi-sequence integration improves lesion characterization	Multi-parametric information captures tissue properties at multiple levels	Protocol variation across scanners; limited public datasets
Histopathological	Flannery et al. [31], Srivastava et al. [32]	1000–10,000	WSI, TMA	Private	Second most common (28.1%); enables cellular-level analysis for Gleason grading	High resolution; gold-standard for cancer diagnosis	Large file sizes; staining variability across institutions
Transrectal Ultrasound	Harmanani et al. [15], Wang et al. [33]	200–1500	TRUS	Private	Used in real-time guidance applications (14.1%); lower cost than MRI	Real-time acquisition; widely available; low cost	Operator-dependent; limited soft-tissue contrast
PSMA PET/CT	Jafari et al. [35], Huang et al. [34]	100–800	PET, CT	Private	Increasing utilization (9.4%); molecular-level characterization	High specificity for cancer detection; whole-body assessment	Radiation exposure; limited spatial resolution for small lesions
Cone-beam CT	Koike et al. [36], Holzschuh et al. [13]	300–1200	CBCT	Private	Used in radiotherapy planning and adaptive treatment	Volumetric imaging during treatment; motion assessment	Lower image quality than diagnostic CT
Challenge Datasets	Multiple studies	50–300	MRI	Public	PROMISE12 and PROSTATEx serve as standardized benchmarks	Enable cross-study comparison; publicly accessible	Limited sample size; single-center acquisition

The analysis reveals dataset heterogeneity challenges, including varying imaging protocols, scanner manufacturers, and acquisition parameters. Multi-institutional studies indicate improved generalizability but face data harmonization complexities. Single-center studies achieve higher image quality consistency while risking population bias.

Annotation approaches vary across applications, from pixel-level segmentation masks to image-level classification labels. Expert radiologist annotations serve as ground truth for most studies, though inter-observer variability remains a concern. Some investigations incorporate multiple expert annotations to quantify labeling uncertainty.

Dataset splitting strategies typically follow 70/15/15 training/validation/test distributions, with patient-level splitting to prevent data leakage. Cross-validation approaches appear in smaller dataset studies to maximize training data utilization. External validation on independent datasets occurs infrequently across the literature.

The temporal distribution of dataset collection spans multiple years, potentially introducing equipment upgrades and protocol changes. Longitudinal patient data appears infrequently, limiting assessment of disease progression modeling capabilities. Multi-modal datasets combining different imaging techniques represent an emerging trend.

### 4.2 Algorithm analysis

#### 4.2.1 Classification

Classification algorithms represent the primary analytical approach for prostate cancer detection across the examined literature. The algorithmic landscape shows evolution from traditional CNN architectures toward transformer-based models and hybrid implementations. Table 2 summarizes the classification algorithms identified across the reviewed studies, including representative publications, main findings, strengths, and limitations.

**Table 2:** Classification algorithms across reviewed studies.

Algorithm	Representative Studies	Main Findings	Strengths	Limitations
ResNet	Rippa et al. [28], Chandrasekhara et al. [37], Liu et al. [16], Sarates & Ozbay [38], Balaha et al. [39]	Most frequently used architecture; residual connections enable training of deeper networks	Training stability; effective transfer learning; strong baseline performance	Computational overhead in deeper variants (ResNet-152 +)
Vision Transformer	Liu et al. [16], Harmanani et al. [15], Li et al. [19], Santhirasekaram et al. [29], Abdelhalim et al. [40]	2–5% accuracy improvement over CNN baselines on large datasets; captures global context	Global feature modeling via self-attention; scalable architecture	Requires large training datasets; high computational cost
VGG	Sarates & Ozbay [38], Ebin et al. [41], Arjmandi et al. [42], Chandrasekhara et al. [37]	Effective for transfer learning with ImageNet pre-training; intuitive architecture	Simple sequential design; well-studied feature representations	High memory consumption; large parameter count
DenseNet	Flannery et al. [31], Sarates & Ozbay [38], Emegano et al. [30], Jusman et al. [43]	Superior performance in histopathological classification; parameter efficient	Dense connections improve gradient flow; feature reuse reduces parameters	Memory constraints during backpropagation in deeper networks
Xception	Sarates & Ozbay [38], Jusman et al. [43], Wei et al. [44], Ebin et al. [41]	Effective in multi-class classification; computational efficiency via depthwise separable convolutions	Reduced parameter count; separates spatial and channel learning	May underperform on tasks requiring fine-grained spatial features
Inception	Sarates & Ozbay [38], Jusman et al. [43], Chandrasekhara et al. [37]	Multi-scale feature extraction through parallel convolution pathways	Captures features at different receptive field sizes simultaneously	Complex architecture; higher training time
ConvNeXT	Rippa et al. [28], Emegano et al. [30],	Competitive with Vision Transformers while retaining CNN computational advantages	Modern design incorporating transformer principles; efficient training	Relatively new; limited medical imaging benchmarks
Swin Transformer	Li et al. [19], Koike et al. [36], Sang et al. [20], Emegano et al. [30]	Hierarchical feature representation through shifted windows; efficient self-attention	Linear complexity with image size; multi-scale features	Requires careful window size selection; complex implementation

ResNet architectures appear most frequently across the reviewed studies, with applications spanning histopathological analysis [28], [37] and MRI-based detection [16]. The residual connection mechanism addresses gradient vanishing problems in deeper network implementations.

Vision Transformer models show increasing adoption for medical imaging applications [15], [16], [29]. The self-attention mechanism enables global feature relationships while maintaining spatial awareness through positional encoding. Comparative evaluations indicate Vision Transformers achieving 2–5% accuracy improvements over CNN baselines for large-scale datasets.

VGG architectures maintain relevance for transfer learning applications, particularly when pre-trained weights from ImageNet provide initialization [38], [41]. The sequential convolutional block design supports feature hierarchy learning, though computational requirements limit scalability.

DenseNet models exhibit superior performance in histopathological classification tasks [30], [31], leveraging dense connections between layers for improved gradient flow. The feature concatenation mechanism enables parameter efficiency while maintaining representational capacity.

Xception architectures show effectiveness in multi-class prostate cancer classification [38], [44], utilizing depthwise separable convolutions for computational efficiency. The architectural design separates spatial and channel-wise feature learning, reducing parameter counts.

Inception models appear in specialized applications requiring multi-scale feature extraction [43]. The parallel convolution pathway design captures features at different receptive field sizes simultaneously. Performance analysis indicates Inception variants perform well in heterogeneous imaging datasets.

ConvNeXT architectures represent modern CNN implementations incorporating transformer design principles [28], [30]. The modernized residual blocks show competitive performance against Vision Transformers while maintaining CNN computational advantages.

ResNet architectures provide training stability through residual connections but carry computational overhead in deeper implementations. Vision Transformers perform well in global feature modeling yet require substantial data augmentation strategies for medical imaging applications. VGG models offer intuitive architectures but involve high memory consumption. ConvNeXT models balance performance and efficiency for moderate-sized datasets.

DenseNet implementations achieve parameter efficiency through feature reuse but encounter memory constraints during backpropagation in deeper networks. Xception architectures reduce computational complexity while maintaining performance but may underperform in tasks requiring fine-grained spatial features.

Vision Transformer architectures are recommended for large-scale datasets with adequate computational resources, while ConvNeXT models provide optimal balance for moderate-sized datasets. ResNet implementations remain suitable for transfer learning scenarios with limited training data. Hybrid approaches combining CNN spatial processing with transformer attention represent a promising direction.

#### 4.2.2 Object detection

Object detection algorithms enable automated localization and classification of anatomical structures and pathological regions within prostate imaging. The reviewed literature presents diverse algorithmic approaches ranging from traditional two-stage detectors to modern one-stage implementations. Table 3 presents the object detection and segmentation algorithms across the reviewed literature.

**Table 3:** Object detection algorithms across reviewed studies.

Algorithm	Representative Studies	Main Findings	Strengths	Limitations
U-Net	Jiao et al. [14], Holzschuh et al. [13], Koike et al. [36], Hampole et al. [45], Sang et al. [20], Li et al. [19]	Dominant architecture for prostate segmentation across modalities; encoder-decoder with skip connections	Precise boundary delineation; preserves spatial information; well-suited for dense prediction	Difficulty with overlapping structures; high memory requirements
R-CNN	da Silva et al. [18], Wu et al. [17], Shabbir et al. [46], Algohary et al. [47], Gon Park et al. [48]	Two-stage detection achieving mAP 0.72–0.89; region proposal followed by classification	High detection accuracy through refinement; flexible architecture	Computational overhead during inference; slower than single-stage
YOLO	Wu et al. [17], Wiratchawa et al. [49]	Single-stage detection suitable for clinical real-time workflows	Fast inference speed; end-to-end training	Accuracy trade-off for speed; difficulty with small lesions
SegNet	Baldeon-Calisto et al. [22]	Encoder-decoder segmentation with pooling indices for upsampling	Memory efficient upsampling; preserves boundary information	Limited receptive field; less accurate than U-Net variants
GAN	Nimitha & Ameer [50], Sultana et al. [51]	Synthetic data generation for augmentation; domain adaptation	Addresses data scarcity; enables cross-domain transfer	Training instability; mode collapse risk
EfficientDet	Wu et al. [17], Wiratchawa et al. [49]	Balanced performance-efficiency through compound scaling	Optimizes depth, width, and resolution jointly	Complex scaling methodology; limited prostate-specific benchmarks
NASNet	Chaddad et al. [52], Haritha et al. [53]	Neural architecture search-derived topology for automated design	Architecture optimized via search; reduces manual design	High search cost; may overfit to search dataset

U-Net architectures dominate prostate segmentation applications across multiple imaging modalities [13], [14], [36], [45]. The encoder-decoder structure with skip connections enables precise boundary delineation while preserving spatial information.

R-CNN family algorithms appear across lesion detection applications [17], [18]. The region proposal mechanism enables candidate region identification followed by classification refinement. Two-stage detection approaches achieve mean average precision (mAP) values between 0.72 and 0.89 across different studies.

YOLO implementations show effectiveness in real-time detection scenarios [17], [49]. The single-stage detection paradigm eliminates region proposal requirements, achieving inference speeds suitable for clinical workflows.

EfficientDet architectures provide balanced performance-efficiency trade-offs [17]. The compound scaling methodology optimizes network depth, width, and resolution simultaneously.

U-Net implementations perform well in dense prediction tasks requiring pixel-level accuracy but encounter difficulties with overlapping structures. The skip connection mechanism preserves fine-grained spatial information while the bottleneck design captures global context. Recent modifications incorporating attention mechanisms address global context limitations.

R-CNN architectures provide high detection accuracy through two-stage refinement but impose computational penalties during inference. YOLO models achieve real-time performance through single-pass detection but sacrifice accuracy for speed in complex scenarios.

U-Net architectures provide superior segmentation accuracy through dense connections and skip pathways but require considerable memory resources during training. R-CNN implementations offer adaptable detection capabilities with good accuracy-speed balance yet face difficulties with real-time applications. YOLO variants achieve fastest inference times while requiring careful attention to small lesion detection.

Hybrid approaches combining U-Net segmentation accuracy with YOLO detection speed present opportunities for next-generation algorithms. Attention-enhanced U-Net variants address global context limitations while maintaining spatial precision. Transformer-based detection methods may provide improved performance through cross-attention between encoder and decoder stages.

### 4.3 Hyperparameter optimization

Hyperparameter optimization strategies across CNN-based prostate cancer detection studies reveal diverse approaches ranging from manual tuning to automated search algorithms. The selection of optimization methodologies impacts model performance, training efficiency, and generalization capability across clinical applications. Table 4 summarizes hyperparameter optimization methods employed across the reviewed studies.

**Table 4:** Hyperparameter optimization methods across reviewed studies.

Optimization Method	Representative Studies	Main Findings	Strengths	Limitations
Genetic Algorithm	Azzeh et al. [21], Singh et al. [54], Das et al. [55]	Most frequently used evolutionary approach; population-based search through selection, crossover, mutation	Superior exploration of large parameter spaces; parallelizable	High computational cost; requires many function evaluations
Bayesian Optimization	Baldeon-Calisto et al. [22], Singh et al. [54]	Probabilistic surrogate model guides efficient search; fewer evaluations needed	Sample-efficient; incorporates prior knowledge; principled uncertainty	May converge prematurely in complex landscapes; surrogate model overhead
Grid Search	Sharma et al., [56]	Exhaustive search over predefined parameter grid; guarantees finding optimum within bounds	Reproducible; simple implementation; guaranteed coverage	Computationally expensive for high-dimensional spaces; curse of dimensionality
Particle Swarm Optimization	Alrefai et al. [57]	Swarm intelligence approach simulating social behavior for parameter navigation	Fast convergence; few hyperparameters to tune	Risk of premature convergence; limited exploration in high dimensions
Random Search	Multiple baseline studies	Stochastic sampling without systematic bias; competitive with grid search	Efficient for high-dimensional spaces; simple to implement	No convergence guarantee; may miss optimal regions

Genetic algorithms emerge as the predominant optimization approach across multiple investigations [21], [54]. The evolutionary optimization paradigm explores parameter space through selection, crossover, and mutation operations.

Bayesian optimization techniques appear in specialized applications requiring efficient parameter space exploration [22], [54]. The probabilistic approach constructs surrogate models to predict parameter performance, concentrating search efforts on promising regions.

Grid search methodologies maintain relevance for systematic parameter exploration across defined ranges [56]. The exhaustive search approach guarantees identification of optimal parameter combinations within specified bounds.

Random search implementations provide baseline optimization performance across multiple studies. The stochastic sampling approach explores parameter space without systematic bias, often achieving comparable results to grid search with reduced computational requirements.

Particle Swarm Optimization (PSO) algorithms appear in cancer classification applications [57]. The swarm intelligence approach simulates social behavior patterns to navigate parameter landscapes.

Performance evaluation across optimization methodologies reveals trade-offs between search efficiency and solution quality. Genetic algorithms provide superior exploration capability but require considerable computational resources for large parameter spaces. Bayesian optimization achieves efficient convergence but may converge prematurely in complex landscapes.

Learning rate optimization appears across all reviewed studies, with typical search ranges spanning 10^−5^ to 10^−1^. Batch size selection influences memory utilization and convergence stability, with common values ranging from 8 to 64 for medical imaging applications. Network depth and width parameters interact non-linearly, requiring joint optimization.

Regularization parameters including dropout rates and weight decay coefficients impact model robustness. Optimization studies typically explore dropout rates between 0.1 and 0.6, while L2 regularization coefficients range from 10^−6^ to 10^−3^. Data augmentation parameters require application-specific tuning.

Automated optimization methods support reproducible model development across different institutions and datasets. Standardized optimization protocols enable fair comparison between algorithmic approaches while reducing human expertise requirements.

Multi-objective optimization approaches balancing accuracy, inference speed, and model complexity represent promising directions. Population-based methods enable parallel evaluation suitable for high-performance computing environments. Hyperparameter transfer learning from similar medical imaging tasks may accelerate optimization for prostate cancer applications.

### 4.4 Explainability

Explainable artificial intelligence (XAI) methodologies provide interpretation mechanisms for CNN-based prostate cancer detection systems, addressing the opaque nature of deep learning models in clinical applications. The reviewed literature shows increasing adoption of interpretation techniques across detection, classification, and segmentation applications. Table 5 presents the explainable AI methods identified across the reviewed literature.

**Table 5:** XAI methods across reviewed studies.

XAI Method	Representative Studies	Main Findings	Strengths	Limitations
SHAP	Li et al. [19], Sang et al. [20], Arjmandi et al. [42], Huang et al. [34], Singh et al. [54]	Most frequently used; localization precision 82–91% compared to ground truth annotations	Theoretically grounded (Shapley values); feature-level contribution quantification	Requires model access for gradient computation; computationally expensive for large models
DiCE	Jiao et al. [14], Holzschuh et al. [13], Gunashekar et al. [23], Li et al. [58], Gon Park et al. [48]	Spatial accuracy 75–85%; generates counterfactual explanations showing minimal changes to alter predictions	Intuitive counterfactual scenarios; temporally stable explanations	Optimization overhead for explanation generation; lower spatial accuracy than SHAP
Grad-CAM	Emegano et al. [30], Ferrero et al. [59],	Gradient-based activation heatmaps for lesion localization; visual overlay on input images	Computationally efficient; applicable to any CNN architecture	Coarse spatial resolution; may highlight irrelevant regions in complex scenes
LIME	Soni et al. [60], Chrystall et al. [61]	Model-agnostic perturbation-based explanations; local decision boundary approximation	Works with any model (black-box); interpretable linear surrogate	Instability across perturbation samples; limited to local explanations
Attention Maps	Wang et al. [33], Zaridis et al. [62]	Inherent interpretability through attention weight visualization in transformer architectures	No additional computation needed; directly reflects model focus	Only available for attention-based models; may not capture full reasoning

SHAP (SHapley Additive exPlanations) implementations appear most frequently across the examined studies [19], [20], [42]. The game-theoretic approach quantifies individual feature contributions to model predictions through coalitional value assignments.

DiCE (Diverse Counterfactual Explanations) methods show effectiveness in medical imaging interpretation tasks [13], [14], [23]. The counterfactual generation mechanism identifies minimal image modifications required to alter model predictions.

Grad-CAM (Gradient-weighted Class Activation Mapping) techniques appear across lesion localization applications [30], [59]. The gradient-based activation approach generates heatmaps highlighting image regions contributing to classification decisions.

LIME (Local Interpretable Model-agnostic Explanations) implementations provide model-agnostic interpretation capabilities [60], [61]. The perturbation-based approach approximates local decision boundaries through simplified models.

Attention map visualization techniques appear in transformer-based implementations [33]. The self-attention mechanism inherently provides interpretation through attention weight distributions.

Quantitative evaluation of explanation quality remains challenging due to subjective interpretation requirements. Faithfulness metrics measure explanation consistency with model behavior, while comprehensibility assessments evaluate human understanding.

Spatial localization accuracy serves as an objective evaluation criterion for vision applications. SHAP implementations achieve localization precision of 82–91% compared to ground truth annotations. DiCE methods show slightly lower spatial accuracy (75–85%) but provide temporally stable explanations.

SHAP methods provide theoretically grounded explanations through axiomatic principles but require model access for gradient computation. DiCE techniques generate intuitive counterfactual scenarios while demanding optimization overhead for explanation discovery. Grad-CAM implementations offer computationally efficient visualization but provide coarse spatial resolution.

Regulatory agencies increasingly require explainable AI systems for medical device approval. The FDA guidance documents stress interpretation transparency for clinical decision support systems. European medical device regulations mandate algorithmic transparency, driving XAI adoption across commercial implementations.

Clinical validation protocols require explanation accuracy assessment against expert annotations. Multi-institutional studies show explanation consistency across different populations and imaging protocols.

Multi-modal explanation methods combining spatial, temporal, and feature-based interpretations may provide fuller understanding. Interactive explanation interfaces enabling clinician feedback could improve explanation quality through iterative refinement. Domain-specific explanation metrics adapted for medical imaging contexts represent an active research area.

Automated explanation evaluation systems could reduce expert annotation requirements while maintaining quality assessment. Explanation personalization based on clinician expertise and preferences may improve clinical acceptance rates. Integration with clinical decision support systems requires explanation delivery formats compatible with existing clinical workflows.

## 5 Discussion

The analysis of CNN applications in prostate cancer detection, classification, and segmentation reveals a rapidly evolving field characterized by architectural innovation, methodological advancement, and increasing clinical integration potential. This discussion synthesizes findings across 320 publications, identifying convergent patterns and persistent challenges.

### 5.1 Challenges

Dataset heterogeneity emerges as the primary obstacle limiting generalizability across CNN implementations. The reviewed literature shows variation in imaging protocols, scanner specifications, and acquisition parameters across institutions [28], [29]. Standardized acquisition protocols and harmonization frameworks are needed to enable cross-institutional model deployment.

Annotation quality and consistency represent persistent challenges across detection applications. Inter-observer variability in expert annotations ranges from 10% to 25% for prostate boundary delineation and 15–35% for lesion characterization [13], [23], [10], [11]. The quality of ground truth annotations directly influences model training outcomes, and studies employing the METRICS framework [25] have shown improved reporting of annotation procedures.

Class imbalance issues affect model training across multiple applications. Healthy tissue samples outnumber pathological cases in most datasets, leading to biased model performance favoring majority classes [30], [31]. Traditional accuracy metrics become misleading under severe imbalance conditions.

Computational resource requirements limit clinical deployment feasibility. Vision Transformer architectures demand considerable memory resources during training, with typical implementations requiring 16–32 GB GPU memory for standard batch sizes [15], [19]. Inference time constraints affect real-time applications.

Regulatory approval pathways remain unclear for AI-based diagnostic systems. Current FDA guidelines require clinical validation protocols that extend development timelines and increase costs. European medical device regulations impose additional transparency requirements that challenge proprietary algorithmic implementations.

### 5.2 Opportunities

Multi-modal integration presents opportunities for improved diagnostic accuracy through complementary information fusion. Combining MRI, ultrasound, and histopathological data sources may provide fuller assessment capabilities exceeding individual modality performance [40].

Federated learning frameworks enable collaborative model development without data sharing requirements [23]. The distributed training paradigm preserves patient privacy while enabling large-scale model development across multiple institutions.

Real-time guidance applications represent emerging clinical opportunities. Intraoperative CNN systems may assist surgical procedures through automated tissue identification and boundary delineation [48], [63]. Biopsy guidance systems could improve sampling accuracy through targeted lesion localization.

Personalized medicine integration offers opportunities for tailored treatment approaches. CNN-based biomarker extraction may identify patient subgroups responding differently to treatment protocols [21]. Predictive models could estimate treatment response probabilities based on imaging phenotypes.

Transfer learning acceleration reduces development time and computational requirements for new applications. Pre-trained models from large-scale natural image datasets provide initialization for medical imaging tasks, achieving substantially faster convergence compared to random initialization [38].

Automated reporting systems present opportunities for standardized clinical documentation. CNN-based lesion detection combined with natural language processing could generate structured radiology reports automatically [64].

### 5.3 Future directions

Architectural evolution toward hybrid models combining CNN spatial processing with transformer global attention mechanisms represents a promising research direction. ConvNeXT implementations show competitive performance while maintaining computational efficiency compared to pure transformer architectures.

Uncertainty quantification integration addresses confidence estimation requirements for clinical deployment. Bayesian neural networks provide prediction confidence intervals enabling clinical risk assessment [65]. Monte Carlo dropout techniques estimate model uncertainty without architectural modifications.

Longitudinal analysis capabilities represent emerging research opportunities. Temporal modeling of disease progression could enable predictive diagnostics and treatment monitoring [66]. Sequential imaging analysis may identify subtle changes indicating treatment response or disease progression.

Domain adaptation methodologies address generalization challenges across different populations and imaging protocols. Adversarial training approaches minimize domain-specific features while preserving diagnostic information.

Automated quality control systems represent practical clinical needs. CNN-based image quality assessment could identify suboptimal acquisitions requiring repeat scanning [67]. Motion artifact detection systems may improve diagnostic confidence through automatic quality scoring.

Interpretability advancement through neurosymbolic approaches may bridge the gap between statistical learning and clinical reasoning. Integration of anatomical knowledge graphs with CNN features could provide structured explanations aligned with medical knowledge.

Edge deployment optimization addresses resource constraints in clinical environments. Model compression techniques including quantization, pruning, and distillation may enable deployment on resource-limited hardware while maintaining diagnostic accuracy [33].

Standardization efforts across the medical imaging AI community could accelerate clinical translation. Benchmark dataset development provides standardized evaluation protocols enabling fair algorithmic comparison. The adoption of reporting frameworks such as METRICS [25] may improve study reproducibility and cross-study comparisons.

Integration with genomic and proteomic data sources presents opportunities for broader disease characterization. Multi-omics fusion approaches may identify novel biomarkers invisible to imaging alone [54]. Correlation analysis between imaging phenotypes and molecular signatures could enable non-invasive disease subtyping.

## 6 Conclusion

This systematic review of 320 publications from 2020 to 2025 reveals that CNN architectures, particularly Vision Transformers for classification and U-Net variants for segmentation, have demonstrated performance levels approaching clinical utility for prostate cancer detection, classification, and segmentation across MRI, histopathology, ultrasound, and PET imaging modalities. Persistent challenges in dataset standardization, regulatory approval pathways, and cross-institutional generalizability require coordinated efforts through multicentric collaborations, federated learning frameworks, and adoption of standardized quality reporting tools such as METRICS. Clinical translation depends on addressing these barriers while advancing real-time deployment capabilities, uncertainty quantification, and explainable AI methodologies to build clinician confidence in automated diagnostic support systems.

## CRediT authorship contribution statement

**Burak Gülmez:** Writing – original draft, Writing – review & editing, Visualization, Validation, Methodology, Investigation, Formal analysis, Data curation, Conceptualization.

## Ethical Statement

Not applicable. This study is a systematic review of published literature and did not involve human subjects, clinical trials, or animal experiments. No ethical approval was required.

## Declaration of Generative AI and AI-assisted technologies in the writing process

Claude Sonnet 4.5 was used for grammar checking and academic style refinement.

## Funding

No funding received.

## Declaration of Competing Interest

The author declares that he has no known competing financial interests or personal relationships that could have appeared to influence the work reported in this paper.

## Data Availability

No datasets were generated or analyzed during the current study.
